# Screening of Extended Family Members of Thalassemia Major Children as a Thalassemia Preventive Strategy

**DOI:** 10.4314/ejhs.v32i6.18

**Published:** 2022-11

**Authors:** Naresh Dattatraya Sonkawade, Aarti Avinash Kinikar, Rajesh K Kulkarni, Rahul M Dawre, Chhaya T Valvi, Pragathi A Kamath

**Affiliations:** 1 Department of Paediatrics, B.J. Government Medical College, Pune, India

**Keywords:** Thalassemia carrier, HPLC, NESTROFT, Extended Family Members

## Abstract

**Background:**

Thalassemia is considered as the most common single gene disorder worldwide. Preventive measures include identification of thalassemia carriers (traits) through screening, genetic counselling and prenatal diagnosis to reduce the incidence. This study aims at estimating the prevalence of carrier status detection among the extended family members of children having thalassemia major so as to use it as a screening prevention strategy with appropriate counselling.

**Methods:**

This cross-sectional study was conducted in thalassemia unit of Pediatric Department of a tertiary care teaching hospital over a period of 18 months. Blood samples were collected from 117 extended family members (EFM) of 23 children with thalassemia major to carry out investigations such as Complete Blood Counts (CBC), Naked Eye Single Tube Red Cell Osmotic Fragility Test (NESTROFT), Reticulocyte count, High Performance Liquid Chromatography(HPLC) and serum ferritin. Reports were analysed to find out the prevalence of carriers.

**Results:**

Among 117 EFM, 62 (52.9%) were males while 55(47.1%) were females. Mean age distribution in this study was 16.49 years (8.5). Prevalence of thalassemia trait (carrier) was 35%. NESTROFT test was positive in 57(48.7%) participants. The binary logistic regression found only positive NESTROFT test as a predictor (adjusted OR=0.022, P=0.001) of having raised HbA2 (HbA2≥3.5 %).

**Conclusion:**

Screening of thalassemia carrier by targeting extended family members of thalassemia major children could yield more carrier cases and targeted counselling could help effectively in decreasing the number of children born with thalassemia major. This strategy could be included in future plan of national prevention programme for thalassemia.

## Introduction

Thalassemia is considered as the most common single gene disorder worldwide with high prevalence in the Mediterranean basin through Middle East, Indian subcontinent, Burma, Southeast Asia and Pacific Islands. Previous studies have shown that the overall prevalence of β-thalassemia carriers is 3–4 % with an estimate of around 8,000 to 10,000 new births every year (1). The prevalence of thalassemia carrier in different regions, castes and tribal communities in India ranges from 1.5 to 37.9% (2,3). According to the Thalassemia International Federation, over 3,30,000 diseased with blood transfusion disorder are born annually, of which 83% are sickle cell disorders and 17% have thalassaemia (around 56,000)(4). Although thalassemia major children manifest clinically in infancy, many children with thalassemia intermediate and thalassemia trait will carry heterozygous genetics to increase the possibility of major cases in future generations.

The change in the incidence of thalassemia in recent years is different in some countries such as Cyprus and Greece, mainly due to effective implementation of prevention programs. Countries like Sardinia have seen a decline in the birth rate of thalassemia children from 1: 250 to 1: 4000 over the years (5). Global screening and preventive programme use one or more of the methods, such as screening of high risk population, school or college students, extended and immediate family members, newborns, premarital and antenatal diagnostic screening (6,7,8,9,10,11,12,13,14).

An excellent feasible solution is to identify thalassemia carriers through screening, provide genetic counselling and prenatal diagnosis to reduce the birth rate of affected infants. Screening methods such as blood indices and NESTROFT are being studied for utility and feasibility in diagnosing thalassemia carriers, but NESTROFT test has some limitations such as low specificity, some technical and quality errors like use of low-quality water, inadequate dilution and frequent change of testing technician giving false reports. Thalassemia screening studies have been conducted in various population groups such as school children, college students and pregnant women. All these studies have not proven to be cost- effective methods for large scale screening.

The diagnosis of thalassemia carriers in extended family members serves as a significant preventive strategy who are at an increased genetic risk. Therefore, this extended family member screening study may provide an alternative to general population screening to identify current and future couples at risk for fathering affected children (15). The present study was conducted to estimate the prevalence of thalassemia carrier status among extended family members of children diagnosed with thalassemia major undergoing regular blood transfusions in a thalassemia day care centre of a tertiary care teaching hospital.

## Materials and Methods

This cross-sectional, observational study was conducted over a period of 18 months in the thalassemia ward of a referral hospital and tertiary care institute. Although the calculated sample size was 66, considering the prevalence of 78.17% carriers after screening extended family members by Thamhankar et al and 10 as a precision, we enrolled 117 study participants as extended family members(EFM) aged 6 months to 30 years(16). Extended family members (EFM) were defined as paternal and maternal uncles, aunts, and first cousins of thalassemia major children(17). We excluded those who have completed their families and older than 30, who have less chances of marrying and giving birth to a child. The clinical history of EFMs was recorded on case record form, and information regarding history of blood transfusions or major blood tests performed in the past was noted. This study was approved by the Institutional Ethical Committee. Subjects were enrolled in the study after receiving written informed consent.

Blood sample of 10 ml was collected from each member for complete blood count (CBC), peripheral blood smear (PBS), reticulocyte count, naked eye single tube red cell osmotic fragility test (NESTROFT), Haemoglobin (Hb) A2 and Serum ferritin levels. CBC was done using an automated cell counter (Sysmex XP-100). Haemoglobin ≤12 gm/dl and MCV <74 fl were considered cut off for analysis in the present study. A peripheral blood smear was prepared from the collected EDTA sample and the morphology of red blood cell was examined. The reticulocyte count was estimated using a manual method with methylene blue staining. NESTROFT was performed by collecting 20 µl of whole blood in EDTA, pipetting into a glass test tube (100 × 10 mm) containing 4 ml of 0.36% buffered saline, shaking the tube and then kept at room temperature for 30 minutes. The tube was shaken again and immediately held in front of a piece of paper with text. If the words on the paper were clearly visible through the tube, the test was labelled as negative; whereas if the words were not clearly visible then labelled as positive. High performance liquid chromatography (HPLC) in BIO RAD VARIANT was used to estimate HbA2 of study members. Reports with an HbA2 of ≥3.5% were diagnosed as thalassemia carriers and <3.5% as normal. Serum separated from the samples was processed with VIDAS serum analyser to estimate serum ferritin.

Primary data was collected and tabulated in Microsoft Excel 2010 spreadsheet. Statistical analysis was performed using IBM SPSS Statistics Version 20. Categorical variables were described as frequency and percentage. Continuous parameters were described as mean and standard deviation. Binary correlation between HbA2 and hematological markers was performed using Pearson's correlation coefficient and scatter plots were used to display the patterns. Univariate and multivariate binary logistics regression was performed to find the independent risk (Odd's ratio) and modified odd's ratio respectively for the occurrence of the dependent variable i.e. Raised HbA2 (HbA2≥3.5%). In this study, p value <0.05 was considered significant and <0.001 was considered highly significant.

## Results

The present study enrolled 117 participants who were extended family members of 23 index cases (thalassemia major). The distribution of study participants with respect to their index cases is shown in Table 1, indicating that a mean number of 5.08 EFMs were studied. We observed that 73(62.4%) study participants were from maternal side of the index cases, while 44(37.6%) were from the paternal side. The gender distribution was 62 (52.9%) males and 55(47.1%) females. The mean age distribution was 16.5 years (8.5), of which 64(54.7%) of the subjects were in the range of 18 to 30 years, which is considered the premarital age range in which genetic counselling helps to decrease the incidence of thalassemia in society.

**Table 1:** Index case wise distribution of Extended Family Members

Index case code	Total No of EFM (n)	Maternal side EFM(n)	Paternal side EFM(n)	Male(n)	Female(n)	Mean of age (±SD)	Total no EFM with HbA2≥3.5% (%)
1	6	3	3	1	5	11.2 (±5.6)	2 (33.3)
2	10	0	10	5	5	15 (±7.5)	3 (30)
3	3	0	3	0	3	15.3 (±8.1)	1 (33.3)
4	2	2	0	1	1	4.1 (±1.7)	1 (50)
5	4	4	0	2	2	27 (±7.9)	2 (50)
6	1	1	0	1	0	25 (±0)	0 (0)
7	5	5	0	2	3	13 (±8.9)	1 (20)
8	5	5	0	3	2	20 (±5.2)	2 (40)
9	4	4	0	1	3	14.8(±8.2)	0 (0)
10	8	8	0	2	6	13.9(±10.2)	3 (37.5)
11	10	9	1	6	4	18 (±8.4)	5 (50)
12	8	5	3	5	3	17.5 (±8.3)	1 (12.5)
13	10	3	7	4	6	17.4 (±9.8)	3 (30)
14	4	0	4	2	2	9.3 (±2.7)	2 (50)
15	7	5	2	5	2	16.3 (±3.3)	2 (28.6)
16	2	0	2	1	1	15 (±11)	1 (50)
17	1	1	0	0	1	20 (±0)	1 (100)
18	3	3	0	2	1	9.7 (±7.3)	2 (66.7)
19	4	1	3	2	2	6.5 (±1.8)	3 (75)
20	7	3	4	6	1	16.6 (±8.5)	2 (28.6)
21	2	2	0	2	0	23.5 (±9.3)	1 (50)
22	8	7	1	6	2	20.2(±9)	3 (37.5)
23	3	2	1	3	0	25 (±5.3)	0 (0)
Total	117	73	44	62	55		

Analysis of laboratory data showed that 49 (41.9%) were anaemic i.e. Hb<12 gm% and 59(50%) with MCV < 74 fl. HPLC analysis showed that 41(35%) had HbA2≥3.5%. Amongst 117 participants, 57 (48.7%) had positive NESTROFT test and 60 (51. 3%) had negative. Haemoglobin ≤ 12 g/dl, MCV < 74 fl, MCH < 25 pg, MCHC<33g/dl, the presence of aniso poikilocytosis in the peripheral smear and positive NESTROFT test were found to be the risk factors with highly significant independent risk of having raised HbA2 (HbA2≥3.5 %), RBC<4.5×10^12^/ L, Haematocrit<40% and RDW ≥18% were also found to be significant risk factors.

**Figure 1:**
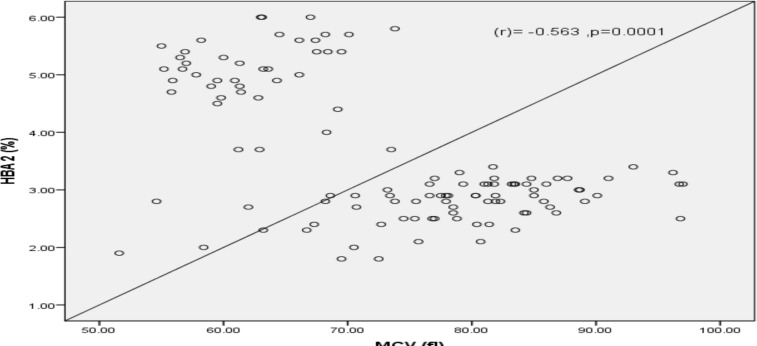
Scatter plot showing correlation between HbA2 and MCV

**Figure 2:**
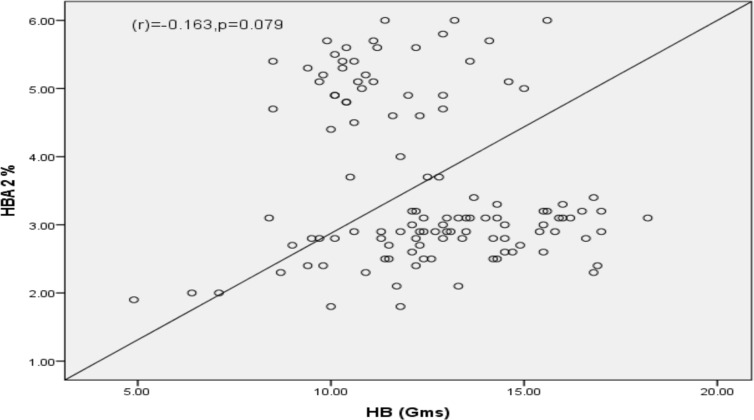
Scatter plot showing correlation between HbA2 and Hb

Binary logistic regression found only positive NESTROFT test as a predictor (adjusted OR=0.022, P=0.001) for elevated HbA2 (HbA2≥3.5%). Other factors were not found to be predictors of elevated HbA2 in the EFMs. (Table 2) A strong negative and highly significant correlation was found between HbA2 and MCV values (r = -0.563)(p=0.0001). (Figure 1) There was a non-significant negative correlation between HbA2 and Hb (r = -0.163) (p=0.079) (Figure 2). Twenty-six study participants had serum ferritin level less than 10 ng/dl, indicating iron deficiency. Among these, 20 subjects had normal HbA2 levels.

**Table 2:** Univariate and Multivariate analysis for risk estimation of abnormal lab parameters to predict the occurrence of HbA2 (HbA2.3.5 %) among EFM of thalassemia index cases

Abnormal Lab Parameters	Raised HbA 2 (HbA2≥3.5) (N=41) n(%)	Normal HbA2 (HbA2< 3.5) (N=76) n (%)	Odds ratio	p Value	Adjusted Odd's Ratio	p Value
Haemoglobin ≤ 12 g/dl(N=49)	27 (65.9)	22 (28.9)	4.73	0.0001	1.89	0.522
MCV < 74 fl (N=59)	41 (100)	18 (23.7)	262.5	0.0001	0.001	0.997
MCH < 25 pg (N=57)	40 (97.6)	17 (22.4)	138.8	0.0001	0.214	0.33
MCHC<33g/dl (N=57)	36 (87.8)	21 (27.6)	18.8	0.0001	1.05	0.95
RBC<4.5×1012/ L(N=14)	0 (0)	14 (18.4)	0.05	0.04	NA	NA
Haematocrit<40% (N=77)	32 (78)	45 (59.2)	2.45	0.04	NA	NA
RDW ≥18% (N=21)	12 (29.3)	9 (11.8)	3.08	0.024	NA	NA
Peripheral Blood Smearaniso poikilocytosis) (N=51)	30 (73.2)	21 (27.6)	7.1	0.0001	0.185	0.063
NESTROFT positive (N=57)	35 (85.4)	22 (28.9)	14.3	0.0001	0.022	0.001
Serum ferritin <10 ng/dl (N=26)	6 (14.6)	20 (26.3)	0.48	0.169	0.185	0.85

p-value considered significant at < 0.05

## Discussion

The present study estimated 35% thalassemia carrier prevalence by targeting population of extended family members of thalassemia major children, which is significantly higher than the reported prevalence range of 0.7% to 16.8% in other studies with population targeted were different clusters of high school or college students, general population and pregnant mothers (7,8,9,10,11,12,13,14,18). Screening extended family members helps in identifying more number of thalassemia carriers thereby helps in targeted counselling in such a high-risk group compared to other groups previously studied. Similar observations have been made in many previous studies of this type, including those by Ahmed et al (19) from Pakistan in 2002, which recorded a prevalence of 31% among 10 index cases. Similarly, Gorakshakar et al (20) reported a prevalence of 21.9% among 691 subjects from Mumbai in 2009, while Tamhankar et al (16) documented a prevalence of 78.2% in 2009, which was the highest of any study. Saquib et al(21) showed a prevalence of 62.2% in a study from Karachi. The data reported by Susanah S et al a study conducted in Indonesia in 2022 estimated the prevalence of 42.7% among EFM of thalassemia major children. El-Shanshory MR et al recorded a prevalence of 35.8 % in 2021, Sharma G et al recorded a prevalence of 48.8% in India in 2016, Husna N et al recorded 44.2% prevalence in 2017 (22,23,24,25). These studies showed higher prevalence as compared to our study probably due to geographical or community specific distribution of thalassemia carrier status.

Extended family members were not equally distributed in the present study as maternal side EFMs outnumbered paternal side, probably because in developing and gender biased countries, people still consider mother and her genes as the sole causative factor for inherited disorder and family members on paternal side blame maternal side for the disease. Therefore, strengthening genetic counselling should be a priority to reduce preventable inherited disorders like thalassemia.

Iron deficiency anaemia with low serum ferritin level can show reduced amount of HbA2,(26) thereby increasing the possibility of missing thalassemia carrier status. Serum ferritin level of less than 10 ng/dl was observed in 20 (17%) study subjects i.e. having iron deficiency anaemia with simultaneous HbA2<3.5 %, so possibility of thalassemia carrier in these study 20 subjects is high which needs to be rechecked after correction of the iron deficiency. Saquib et al(21) had similar observations and they offered six months of iron treatment and advised for repeat HbA2. This highlights the importance of serum ferritin assay in the screening programme of thalassemia carrier where iron deficiency anemia is very common in the young as well as adults. Some limitations of the study are our inability to follow up study participants with iron deficiency to repeat their HPLC, could not enroll uniform number of subjects from 23 index cases, molecular or DNA analysis confirmatory test were not performed in these study subjects owing to financial constraints.

Prevalence of thalassemia carrier was high (35%) among EFM of thalassemia major children in the present study. Targeting extended family members of thalassemia major affected children could yield more carrier cases detection. It can be a cost-effective strategy and may help in preparation of national programme for prevention of thalassemia. Iron deficiency anemia should be ruled out in all the cases screened for thalassemia carrier status as it is more common in developing countries.
